# The factors affecting effectiveness of treatment in phages therapy

**DOI:** 10.3389/fmicb.2014.00051

**Published:** 2014-02-18

**Authors:** Mai Huong Ly-Chatain

**Affiliations:** Laboratoire Bioengénierie et Dynamiques Microbiennes aux Interfaces Alimentaires (BioDymia) ISARA-LyonLyon, France

**Keywords:** phage therapy, antibiotic resistance, effectiveness, pathogenic bacteria, Phage

## Abstract

In recent years, the use of lytic bacteriophages as antimicrobial agents controlling pathogenic bacteria has appeared as a promising new alternative strategy in the face of growing antibiotic resistance which has caused problems in many fields including medicine, veterinary medicine, and aquaculture. The use of bacteriophages has numerous advantages over traditional antimicrobials. The effectiveness of phage applications in fighting against pathogenic bacteria depends on several factors such as the bacteriophages/target bacteria ratio, the mode and moment of treatment, environmental conditions (pH, temperature...), the neutralization of phage and accessibility to target bacteria, amongst others. This report presents these factors and the challenges involved in developing phage therapy applications.

## INTRODUCTION

Bacteriophages (phages) are viruses that only infect bacteria. They are approximately 50 times smaller than bacteria (20–200 nm) and ubiquitous in the soil, water, and several food products (meat, vegetables, dairy products...; Kutter and Sulakvelidze, 2005). The “virulent” and “temperate” phages differ in their mode of action. The first step of the phage infection is adsorption of the phage particle to the bacterial cell wall by specific interactions between viral surface proteins and host cell receptors. After entering the bacterial cell, the virulent phages replicate rapidly to synthesize genome and structural proteins into progeny virions inside the host cell. Finally the new phages escape by rupturing the cell wall which results in the death of the cell. In contrast, temperate phages integrate their genetic material into the chromosome of the host cell, which is replicated along with the host cell genome (prophage). They can, therefore, subsequently emerge inside a new host cell. Only temperate phages which can enter the bacterial genome participate in horizontal gene transfers between bacterial populations. For antibacterial applications, virulent phages which have the ability to rapidly lyse bacterial cells are employed.

The use of bacteriophages to treat bacterial infections was studied prior to the Second World War. These studies were not followed up once antibiotics had been discovered. But in recent years, with the emergence of several bacterial strains multiresistant to antibiotics, research has turned back to bacteriophages. The use of phages to inactivate pathogenic bacteria is seen as an interesting way of replacing antibiotics in human medicine. Indeed, bacteriophages are considered as “intelligent antimicrobials” due to the specificity of their action. They infect the target bacteria without any effect on commensal flora and are naturally eliminated along with the complete eradication of pathogenic bacteria (Kutateladze and Adamia, 2010; Jenny, 2011).

In veterinary medicine, numerous studies have been carried out to combat bacterial diseases and control transmission to humans of the pathogens responsible for foodborne illnesses. For example, the reduction of *Campylobacter* and other pathogenic bacteria contamination by phages has been studied in several publications (Berchieri et al., 1991; Goode et al., 2003; Connerton et al., 2004; Fiorentin et al., 2005; Wagenaar et al., 2005; Higgins et al., 2008). The poultry and medical fields can benefit from these results, in terms of reducing economic losses and improving the overall well-being of consumers.

Bacteriophages have been studied in the agri-food industry in order to detect and control pathogenic bacteria in foodstuffs (Martínez et al., 2008; Guenther et al., 2009; Holck and Berg, 2009; Jamalludeen et al., 2009; Kocharunchitt et al., 2009; Hudson et al., 2010; Bigot et al., 2011; Mahony et al., 2011). The advantage of using lytic phages lies in their high specificity toward the host pathogenic bacterial strains. They do not affect technological flora or the commensal flora of the digestive tract. In addition, bacteriophages do not cause human allergies and nor do they change the structure, odor or flavor of food products (Hagens and Offerhaus, 2008).

Research on the phages has been extensive over the last decade (Pirnay et al., 2012). Many animal models have been available for reliable studies. Research on phages has expanded beyond the laboratory. Phage products have been approved and commercialized. Regularly, approvals have been granted in the USA for commercial phage products. In 2006 the FDA (Food and Drug Administration) approved the use and the preparation of bacteriophages generally recognized as safe (GRAS) as food additives for the control of the pathogenic bacterium *L. monocytogenes* in meat and poultry products. In Europe, the use of Listex^TM^ was also approved in Switzerland for cheese making and has recently been approved for other food types. The Listex^TM^ has also been approved for use in food processing by food standards Australia and New Zealand (FSANZ) in 2012. Several phage products are currently produced on a commercial scale:

### BACTERIOPHAGES ON A COMMERCIAL SCALE

LMP-102^TM^ (Listshield^TM^) produced by Intralytix Inc. (USA) includes six bacteriophages This product occurs naturally in the environment and is used to control *L. monocytogenes* in ready-to-eat meat and poultry products before packaging. Other similar products such as ECP-100^TM^ (Ecoshield^TM^) target *E. coli O157:H7* in ground beef, fruits, and vegetables.

Listex^TM^ is a phage preparation derived from phage P100 that targets *L. monocytogenes *by EBI Food Safety (Netherlands). Phage P100 was originally isolated from a wastewater sample taken from a dairy plant in Germany in 1997 (Carlton et al., 2005). In 2011 the company started selling its new phage product, effective against *Salmonella*, branded: SALMONELEX^TM^.

Omnilytics, Inc. (USA) has two products that target bacteria on animal hides prior to slaughter. Both products are termed BacWash^TM^ targeting *Salmonella* and *E. coli O157:H7*. BacWash^TM^ can be applied as a wash, mist or spray directly to the live animal. Future potential uses of the BacWash^TM^ line of products include the treatment of animal holding areas, transportation equipment and containers, and living areas.

Other phage products are currently commercialized and developed by several companies such as AgriPhage ^TM^ of Omnilyics (USA) targets *Xanthomonas campestris *or* Pseudomonas syringae. *BioPhage-PA is product of AmpliPhi biosciences Corp (UK) for the treatment against *Pseudomonas aeruginosa* in chronic ear disease and topical injection. Viridax ^TM^ is being developed to treat the *S. aureus* by Viridax Company (USA).

**FIGURE 1:**
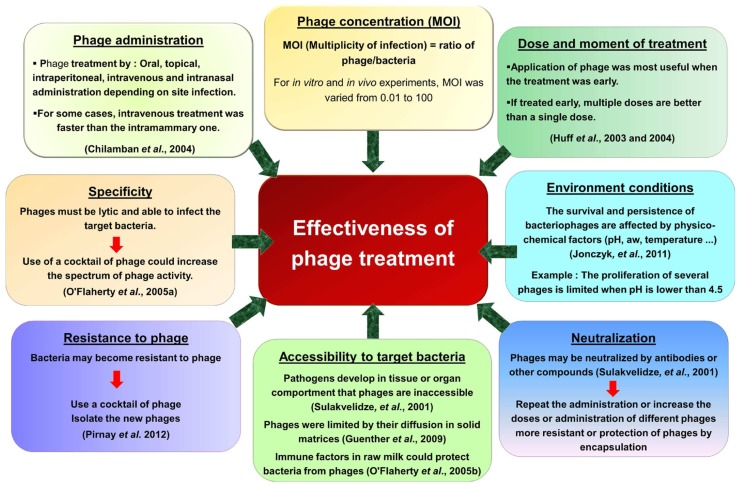
**Factors affecting the effectiveness of phage use against pathogenic bacteria**.

Despite this increase in research interest and the production of phage products, the application in phage therapy was not always successful. The effectiveness of phage applications against pathogenic bacteria depends on several factors such as the bacteriophage/bacteria ratio, physico-chemical factors (pH, temperature...), phage neutralization or resistance to phage. Moreover, the data *in vitro* cannot be directly applied to the *in vivo* situation and nor can *in vivo* data for one phage be transferred to another phage. Critical parameters that affect phage therapy are the phage adsorption rate, burst size, the latent period and initial phage dose (Payne et al., 2000; Payne and Jansen, 2001). Another critical parameter is the clearance rate of the phage particles from the body fluids by the reticuloendothelial system or the phage-neutralizing antibody phenomena. Some key factors are presented in **Figure 1**.

#### Phages/bacteria ratio

The use of bacteriophages against pathogenic bacteria has been studied using two different approaches, one passive, the other active (Gill, 2010). In the case of the passive approach, the bacteriophages are added into the system at a level sufficient to ensure that all target bacteria are infected and lysed in a short period of time. On the contrary, active biocontrol relies on the addition of a small amount of phages. Bacterial elimination, in this case, supposes the replication of phages over several generations. The capacity of new replicated phages to access the target bacteria could be weakened by the biochemical and physico-chemical characteristics of the system (the viscosity for example). It appears that the passive treatment is more efficient than the active one.

In virology the bacteriophages/bacteria ratio is explained by the term MOI (multiplicity of infection)which refers to the number of virus that are added per cell during infection. MOIis used only in fluid systems with high numbers of host cells. In* in vitro* and *in vivo* experiments on phage against bacteria, the MOI comprised between 0.01 and 100 are classically used. Most often, MOI is 100 to ensure there is enough phage in the media. However, not all phages replicate or survive in the same way. It is important to determine the replication lytic cycles and the resistance of phages in respect of environmental conditions.

#### Environmental conditions and phage resistance

The survival and persistence of bacteriophages are affected by physico-chemical factors (pH, ions, temperature...; Jonczyk et al., 2011). The phage population is generally stable in relation to external factors. Some phages can be stored for a long period in neutral pH (6 to 8) in solution or in dried form (Jonczyk et al., 2011). Phage titers generally are decreased slowly with pH. For example, the phage titer of *S. aureus* was reduced 2 log between 4 and 6 h when pH decreased from 6.19 to 5.38 (Garcia et al., 2009). The proliferation of several phages is limited when pH is lower than 4.5, but the risk of pathogenic bacteria food contamination is also generally reduced below pH 4.5. For example, the phage T4 (*Myoviridae* family) is unstable at pH < 5. Phage PM2 (*Corticoviridae* family) loses completly activity after 1 h at pH 5.0 at 37°C. However, in the case of phage oral injection, stomach acid can have a negative impact on the survival of phage which may lead to treatment failure (Watanabe et al., 2007). The latent period is increased when the phages are incubated at refrigeration temperatures. Bacteriophage can survive at high temperatures (40–90°C) and some phages of *Lactococcus* can survive pasteurization (Madera et al., 2004). In the study of MS2 phage stability in different salt solutions the authors showed that the monovalent salts did not influence phage titer (Mylon et al., 2010). According to Langlet et al. (2008) higher ionic strength can increase the aggregation of phage (Langlet et al., 2008).

Besides these environmental conditions, the biochemical composition of the matrix also influences the accessibility of target bacteria.

#### Accessibility to target bacteria

According to Marco et al. (2010) the diffusion of bacteriophages could be impaired or favored depending on the structure and the composition of the matrix and the environmental conditions (Marco et al., 2010). In solid media, the diffusion of bacteriophages could be limited, reducing phage adsorption on bacteria and, consequently, the phage infection capacity. For example, Guenther et al. (2009) have shown that the use of bacteriophages was limited by their diffusion in solid food matrices such as hot dogs, smoked salmon and seafood.

The presence of other compounds could protect bacteria from phages. The phage K is active on numerous strains of *S. aureus,* but was inactive in raw milk which limits its application in bovine mastitis (Gill et al., 2006a). O’Flaherty et al. (2005) suggested that the immune factors present in raw milk prevented phages from gaining access to bacteria. According to Gill et al. (2006b), some proteins in whey may inhibit the adsorption of phage on bacteria.

In phage therapy, the escape of invasive pathogens into closed tissue and organ compartments may block the effective use of bacteriophages, especially if the phage cannot actively follow the bacteria. It is also unclear how effective phages would be in treating diseases caused by intracellular pathogens (e.g., *Salmonella* species), where bacteria multiply primarily inside human cells and are inaccessible to phages (Sulakvelidze, 2005).

In the study of Chibani-Chennoufi et al. (2004) the author showed that the survival of the bacteria in the gut during the phage passage could only be explained by some physiological reasons that prevented phage-induced lysis. The axenic mice were infected with a single *E. coli* strain and then were given phages in drinking water. The phage titers in the stools increased in one day from the 10^5^/mL in the drinking water to 10^10^/ml in the stool while at the same time numbers of *E. coli* in the stools reduced from 10^8^ to 10^4^ CFU/mL and stabilized at 10^5^ CFU/mL during the subsequent days. The bacteria were not completely lysed in the stool although these bacteria are sensitive to the phages. These results suggest that bacteria had resided in gut sites protected from phage (Chibani-Chennoufi et al., 2004).

Phages, unlike many antibiotic molecules, are not diffusible across membranes and must therefore have a method of delivery to reach the target cells. Some researchers believe that the best delivery mechanism may lie in using other non-pathogenic species of bacteria to bring the phage to its pathogenic target (Inal, 2003).

#### Circulation of phage and neutralization of phage by antibodies

To understand further the accessibility of phage to bacteria, some authors have studied how phages circulate but few publications are available on the subject. Some authors suggest that phages get into the bloodstream of laboratory animals (after a single oral dose) within 2 to 4 h and that they are found in the internal organs (liver, spleen, kidney, etc.) in approximately 10 h. Also, data concerning the persistence of administered phages indicate that phages can remain in the human body for relatively prolonged periods of time, i.e., up to several days (Bogovazova et al., 1991, 1992).

In an experimental design where mice were infected with the ϕMR11 lysogen strain, no protection was observed allowing the authors to conclude that a direct bactericidal effect of the phage was the principal determinant of the protective effect rather than any indirect effect such as a phage-stimulated immune response (e.g., production of cytokines; Matsuzaki et al., 2003). Phage and bacterial numbers in the circulation were determined after the infection and showed that the bacterial load was much lower in the blood of phage-treated mice when compared to those that received only bacteria. They also noticed that phage titers in mice infected with bacteria remained higher than the titers in mice that received only the phage. This suggested that the phage had replicated in the infected mice and consumed the bacteria.

Although the phage can circulate well in the blood and in different organs but some authors suggest that the phages may be neutralized by antibodies which hamper phage effectiveness to lyse the targeted bacteria. Geller et al. (1998) have showed that the addition of colostrum in milk contaminated by phage prevented the lysis of starter cultures of *L. lactis*. However, it is not clear how long the antibodies will remain in circulation. According to (Sulakvelidze et al., 2001), the development of neutralizing antibodies should not be a significant obstacle during the initial treatment of acute infections, because the kinetics of phage action are much faster than the host’s production of neutralizing antibodies. Moreover, if phage-neutralizing antibodies are still present at the time the second course of treatment is administered or if a rapid anamnestic immune response occurs before the phages exert their action, it could be envisaged to repeat administration or increase the phage concentration. Another solution to survive the phage neutralization by antibodies would be to use different phages because resistance is different from one phage to another.

Merril et al. (1996) developed an ingenious method to solve this problem. They succeeded in isolating the mutants, whose stability in the blood increased, by repeating the following procedure eight to ten times: (1) administration of phages into the peritoneal cavity of the mouse, (2) recovery of phages from the blood 7–18 h after the injection, (3) multiplication of the recovered phages *in vitro*, and (4) readministration of the proliferated phages to mice. The mutant derived from phages after a long-circulating had capsid protein modified (Merril et al., 1996).

#### Protection of phages

One of the solutions to protect the bacteriophage at the site of infection and during the journey to this site is microencapsulation which is defined as a technology of packaging solids, liquids, or gaseous materials in miniature capsules that can release their contents at controlled rates under specific conditions. Microencapsulation has been applied to enhance the viability of probiotic bacteria during processing and also for targeted delivery to the gastrointestinal tract. The microencapsulation of viruses has been studied as an effective adjuvant system to induce specific immune responses via mucosal routes. Development of oral microencapsulated forms for bacteriophages to treat gastro infection in cattle has been reported (Ma et al., 2008; Dini et al., 2012). These authors have shown that the encapsulation technique enables a large proportion of bacteriophage to remain bioactive in a simulated gastrointestinal tract environment, which indicates that these microspheres may facilitate delivery of therapeutic phage to the gut.

#### Dose and moment of treatment

Another important factor that can modify the effectiveness of phage treatment is single dose versus multiple doses. Several studies have shown that multiple doses are better than a single dose. One study by Huff et al. (2003a,b) found that treating chickens suffering from severe respiratory infections caused by *E. coli* was very helpful in clearing up symptoms. The application of bacteriophage was most useful very soon after the chickens had been exposed to the bacteria and that, if treated early, multiple doses were better than a single dose. Interestingly, if treatment starts later, there is no difference between single or multiple doses, but treatment is still very helpful (Huff et al., 2003a,b).

In a very thorough study, Biswas et al. (2002) performed experiments using a mouse model of vancomycin- resistant *Enterococcus faecium* infection. They showed first that a phage administered intraperitoneally 45 min post-infection was able to rescue mice from *E. faecium* and that the rescue was associated with a significant decrease in bacterial numbers in the blood. They also demonstrated that phage administration up to 5 h post-infection still fully rescued the mice while treatment delayed beyond 5 h rescued only some of the mice (Biswas et al., 2002).

#### Phage administration

One advantage of phage use is the easy administration. Phages can be applied by oral,_topical, intraperitoneal, intravenous, or intranasal administration. The phage preparation and production may be carried out in pathogenic bacterial culture. In this case it must be controlling the toxin or the residues of bacteria culture which may provoke the inflammatory phenomenon (Gill and Hyman, 2010).

Phage therapy has been used for the treatment of a variety of bacterial infections. They can be used in freeze-dried form and turned into pills without materially impacting efficiency. Temperature stability up to 55°C and shelf lives of 14 months have been shown for some types of phages in pill form. Application in liquid form is also possible, stored preferably in refrigerated vials.

Oral administration works better when an antacid is included, as this increases the number of phages surviving passage through the stomach.

Topical administration often involves application to gauzes that are laid on the area to be treated.

In the study of efficacy of bacteriophages for the treatment of infections caused by *Klebsiella ozaenae, K. rhinoscleromatis scleromatis *and* K. pneumonia *(Bogovazova et al., 1991, 1992), the phage preparation was reported to be efficacious in treating experimental infections of mice and guinea pigs. *Klebsiella* polyvalent bacteriophage was administered intraperitoneally, intravenously or intranasally on day 2 after the infection of the animals with *Klebsiella*. The result showed that the bacteriophage introduced intraperitoneally, was effective in the treatment of a generalized *Klebsiella* infection. Other authors Chilamban et al. (2004) have also shown that intravenous inoculation was faster than the intramammary onewhen they studied theefficacy of a specific lytic phage against *S. aureus *in a mice model.**

However, it is difficult to conclude which mode of administration is the most effective. The effectiveness of treatment depends on various factors: the concentration of pathogenic bacteria on the infection site, phage preparation, and the dose applied, medium composition and structure, and environmental conditions...

#### Specificity

Phages specifically infect the host bacteria species. This specificity can limit the effectiveness of phage use. To ensure that the bacteria can be lysed by the phage used, the bacterial strain isolated from the infection site will be tested for its sensitivity to the phage administrated. It is important also to verify if the phage is strongly lytic or not. However, polyvalent phages which can infect several bacteria strains of the same species do exist. The use of polyvalent phage allows the activity spectrum of phages to be increased. The polyvalent phage can be replaced by a cocktail of phages. Briefly, the phage by their specificity can infect only the target bacteria without effect on others bacteria flora, but the specificity may also have an ineffective treatment if the target bacteria are not lysed by the phages administrated. To overcome the problem related to the specificity of phage, several solutions are proposed:

– Isolating the bacteria from the infection site and screening the sensitivity of this bacteria against a panel of bacteriophages.
– Selecting polyvalent bacteriophages with broad cross-strain lytic activity
– Developing a cocktail of phages which could increase the spectrum of activity of the phages against all or most of the strains within a given species of bacterial pathogen.

#### Resistance to phage

As in the case of antibiotics, bacteria can develop resistance to phage, which may hamper the effectiveness of phage treatment. The first step of phage infection to bacteria is adhesion of phage on bacterial surface by surface proteins which act as receptors. If the bacterium loses the phage receptor, they become resistant to phage. Bacteria may also acquire horizontally a restriction-modification system that degrades the nucleic acid of the injected phage. In addition, phage resistance may be caused by a mutation in a gene, the product of which is essential for phage replication or assembly. In any case, the resistance to phage does not cause a problem for phage use or phage therapy because the rate at which bacteria develop resistance to phages is approximately 10-fold lower than to antibiotics (Carlton, 1999). Moreover, this rate can also be partially circumvented by using several phages in one preparation much like using two or more antibiotics simultaneously. When resistance against a given phage occurs, a new phage can be created to target and destroy the new strain. Some protocols on isolation of phage have been mentioned in literature (Garcia et al., 2009; Moineau and Fortier, 2009). The selection and screening a new phage is faster than the development of novel antibiotics which can take up to several years (Sulakvelidze et al., 2001).

## Conclusion

The use of bacteriophages as antimicrobial agents controlling pathogenic bacteria has appeared as a promising new strategy and it seems that phage therapy may provide a good alternative solution to antibiotics. The abundance of phages in the environment highlights their potential use for control of pathogenic bacteria in food and animals. For an effective treatment, bacteriophages should be (1) present in high concentrations, (2) stable over time and in *in vivo* conditions, (3) able to meet the bacteria without any restriction, and (4) able to replicate. For that, some points should be taken into account:

– Use a high level of phage concentration
– Use phage to treat the bacterial infection as soon as possible
– Test the stability of phage in real environmental conditions
– Protect the phage by microencapsulation
– Screen and develop the cocktail of phage lytic which is able to infect many bacterial strains
– Use a polyvalent bacteriophages with broad cross-strain lytic activity or develop a phage cocktail to lyse the majority of bacteria strains and limit the development of resistance to phage

However, several challenges may arise in phage therapy such as:

– phages can be neutralized by antibodies or other components in the matrices
– bacteria may develop on several sites which are inaccessible to phage.

There is a need to develop a model which approaches the *in vivo* conditions to elucidate the factor influence on the infection capacity of phages before *in vivo *application.

## Conflict of Interest Statement

The author declares that the research was conducted in the absence of any commercial or financial relationships that could be construed as a potential conflict of interest.
